# Magnetoelectric properties of bulk 0-3 Fe/BaTiO_3_-composites

**DOI:** 10.1039/d5ra03466c

**Published:** 2025-09-15

**Authors:** Toni Buttlar, Hartmut S. Leipner, Stefan G. Ebbinghaus

**Affiliations:** a Institute of Chemistry, Martin Luther University Halle-Wittenberg Kurt-Mothes-Strasse 2 06120 Halle Germany stefan.ebbinghaus@chemie.uni-halle.de +49-345-5527028 +49-345-5525871; b NorcSi GmbH Weinbergweg 23 06120 Halle Germany

## Abstract

Magnetoelectric 0-3 Fe_*x*_/(BaTiO_3_)_1−*x*_ composites (*x* = 0.1–0.8) were synthesized by reduction of Fe_2_O_3_/BaTiO_3_ pellets in forming gas. In a subsequent sintering step, dense composite ceramics were formed. Depending on the oxygen getter used in the sintering step (carbon or zirconium carbide) a partly iron-doped or undoped ferroelectric barium titanate matrix is obtained, which encloses micrometer-sized ferromagnetic Fe particles. The experimentally determined iron contents derived from Rietveld refinements and magnetic measurements are in good agreement with the nominal ones in the undoped composites. Field dependent magnetoelectric measurements revealed only small differences between the composites with doped and undoped BaTiO_3_ component when the magnetic field and the polarization are oriented parallel to each other. For samples with iron-doped BaTiO_3_, additional third extrema at low fields were found when the field was oriented perpendicular to the electric polarization whereas undoped samples exhibited only two extrema. The largest *α*_ME_ values were measured for the Fe_*x*_/(BaTiO_3_)_1−*x*_ composites with *x* = 0.4 (parallel) and *x* = 0.3 (perpendicular). Based on the integral of *α*_ME_, the magnetostriction of iron and a phenomenological model describing the connection between magnetostriction and the magnetoelectric effect was derived. In temperature-dependent magnetoelectric investigations the low-temperature phase transitions of BaTiO_3_ (tetragonal → orthorhombic → rhombohedral) were detected.

## Abstract 2

Fe/BaTiO_3_ composites were synthesized systematically and the complex field dependence behavior of the magnetoelectric coefficient was investigated.

## Graphical abstract

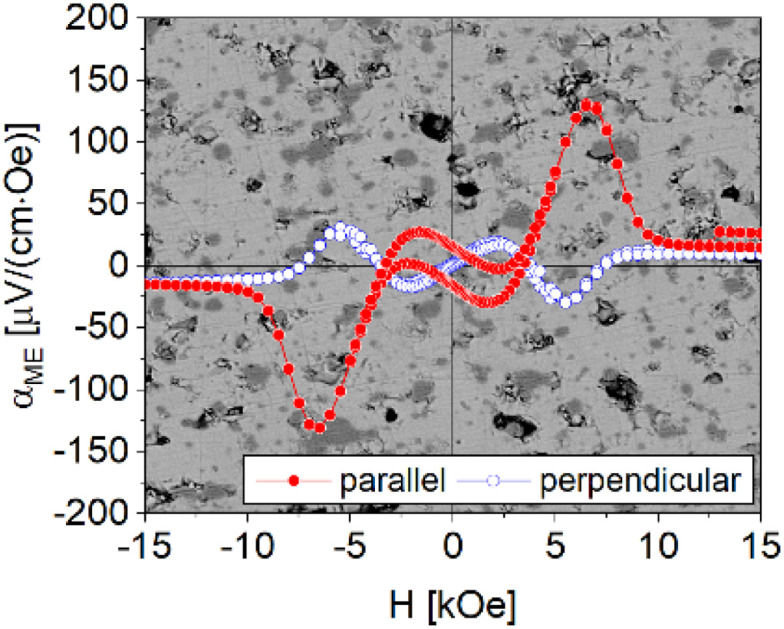

## 1. Introduction

The interaction between the magnetic and electrical properties in multiferroic materials (single component or composites) is denoted as magnetoelectric coupling. In composites, the magnetoelectric effect (ME) is based on the combination of the magnetostriction of a ferro- and ferri-magnetic substance and the piezoelectricity of a ferroelectric material.^1–4^

A possible classification of the magnetoelectric composites is based on the dimensionality of its components. In addition to the well investigated heterolayers (2-2) and particles in a matrix (0-3) other connectivities (*e.g.* fibers in a layer (1-3)) are possible.^5–7^

Besides lead zirconate titanate (PZT) and strontium barium niobate (Sr_0.5_Ba_0.5_Nb_2_O_6_) barium titanate (BTO) is a commonly used component because of its well-examined ferroelectric properties.^2,8–10^ BaTiO_3_ possesses three different ferroelectric structures below its cubic to tetragonal phase transition at *ca.* 393 K.^11,12^ As the three phases show different ferroelectric properties the transitions at *T*_t↔o_ ≈ 273 K (tetragonal to orthorhombic) and *T*_o↔r_ ≈ 183 K (orthorhombic to rhombohedral) can be detected by magnetoelectric measurements as shown in previous studies.^13,14^

Metals (*e.g.* Fe, Co and Ni),^13,14^ alloys (*e.g.* CoFe, Terfenol-D (Tb_1−*x*_Dy_*x*_Fe_2_))^4,15^ and magnetically ordered oxides (*e.g.* ferrite spinels)^5,6,9,10,16^ are possible ferro-respectively ferrimagnetic materials for magnetoelectric composites. Due to the often well-investigated magnetostrictive behavior of the ferromagnetic metals a fundamental understanding of the magnetostrictive influence on the magnetoelectric effect may be achieved. In two proceeding articles we already investigated 0-3 Ni/BaTiO_3_ and Co/BaTiO_3_ composites.^13,14^ Here, we report on related samples containing iron as magnetic component.

Iron is the ferromagnetic 3d-metal with the highest magnetic moment (*M*_S_ = 2.2 *μ*_B_ per atom (ref. 17)) at room temperature and has a Curie temperature of *T*_C_ = 1044 K.^18^ It exhibits a very special magnetostrictive behavior: in small magnetic fields (up to 300 Oe) iron shows a positive magnetostriction whereas with increasing field the magnetostriction changes to negative values.^19^ This change might lead to new magnetoelectric properties, which can be interesting for sensoring or data storage. Noteworthy is also the pressure dependence of the magnetostrictive behavior of iron. After applying and releasing of a tension within the elastic limit, the positive maximum of the magnetostriction vanishes with increasing tension. However, when tensions above the elastic limit are applied and removed an increasing of the positive maximum was observed.^20^ Under permanent applied compressive stress the magnetostriction of iron increases to positive values and the maximum is raised. In contrast, under permanently applied tensile stress a shrinking and vanishing of the maximum and an increasing of the negative saturation magnetostriction values can be observed.^20–22^ Furthermore, in the temperature range from 273 K to 1000 K a change in the sign of the magnetostriction and a maximum in the range 773 and 873 K is found.^20,23^

BaTiO_3_ can be doped with iron up to 1.25 mol%.^24^ This doping changes its dielectric and piezoelectric properties. The cell parameters of Fe-doped barium titanate show a reduced tetragonal distortion and with higher iron contents it becomes less ferroelectric. As a result the magnetoelectric coupling is reduced.^25,26^

Due to the possibility of an oxidation of metallic iron during the synthesis and in turn the potential doping of the BaTiO_3_ matrix of 0-3 Fe/BaTiO_3_ composites have barely been investigated.^27,28^ Aside from the few experimental studies mostly theoretical aspects and laminated heterostructures (2-2) have been investigated.^29–37^

To expand the knowledge of the magnetoelectric behavior of 0-3 metal/BaTiO_3_ composites, we reduced mixtures of Fe_2_O_3_ and BaTiO_3_ followed by a sintering step in flowing nitrogen gas using different oxygen getters to synthesize 0-3 Fe_*x*_/(BaTiO_3_)_1−*x*_ composites with *x* = 0.1–0.8. Besides investigations of the structural influence of a possible Fe-doping *via* X-ray diffraction and SEM/EDX, we analyzed the dielectric, the magnetic and the magnetoelectric properties of the different composites. Special focus was put on the field and the temperature dependence of the magnetoelectric effect.

## 2. Experimental

### 2.1. Sample preparation

The synthesis of Fe_*x*_/(BaTiO_3_)_1−*x*_ (*x* = 0.1–0.8) was performed as described for Ni/(BaTiO_3_) and Co/(BaTiO_3_).^13,14^ A mixture of BaCO_3_ (Solvay, Sabed VL 600, Lot.-Nr. 538320) and TiO_2_ (Venator, TR-HP-2, Lot.-Nr. UOC9410) was grinded with agate balls and isopropanol in polyamide jars in a planetary mill Pulverisette (Fritsch) over night and subsequently calcined at 1373 K for 1 h (heating rate: 5 K min^−1^) in a Nabertherm box furnace (Type LHT 02/17/P310). Stochiometric quantities of Fe_2_O_3_ (Alfa Aesar, Puratronic®) and the synthesized BaTiO_3_ were mixed together with agate balls and isopropanol in agate jars in a planetary mill PM400 (Retsch) for 18 h. Pellets of these mixtures with 6 mm diameter and *ca.* 100 mg weight were pressed applying a force of 2 kN. The reduction of Fe_2_O_3_ to metallic iron was performed in a tube furnace (Elite Thermal System Ltd) at 1073 K in forming gas (Linde, N_2_/H_2_: 90/10; gas flow: 80 ml min^−1^; equilibrium oxygen partial pressure *ca.* 5 × 10^−24^ bar) with a heating rate of 5 K min^−1^ and a dwell of 2 h. Finally, the pellets were sintered in a tube furnace HF-1800 (Crystal Systems Corporation) at 1623 K (heating rate: 2.5 K min^−1^) for 2 h in nitrogen (Linde, 5.0, gas flow: 150 ml min^−1^) with graphite (Fluka) respectively zirconium carbide (ChemPUR) as oxygen getter. The equilibrium oxygen partial pressures were measured with an Oxygen Measuring Module ZR5 from Zirox.

### 2.2. Characterization

X-ray powder diffraction measurements for phase analysis were done at room temperature in the angular range between 15–70° 2*θ* with a step width of 0.01° and counting time of 0.5 s per data point on a Bruker D8-Advance diffractometer (Cu-K_α1,2_ radiation) equipped with a silicon strip LynxEye-detector. SmartLab Studio II from Rigaku Corporation was used to determine the crystallite sizes of iron applying the Scherrer equation on the peak at 44.8. For the determination of the cell parameters of BaTiO_3_ and Fe Rietveld refinements (angular range 15–120° 2*θ*, step width = 0.01°, counting time = 1 s per data point) were carried out. Before the investigation of the pellets their surfaces were polished first with SiC paper (grit 2000) followed by fine-polishing with 2 μm diamond suspension both with a Struers LaboPol 5 polishing device. A Phenom ProX was used for scanning electron microscopy (15 kV, backscattered electron mode) and energy-dispersive X-ray spectroscopy (EDX) measurements applying the ZAF-correction method. The dispersion of grain sizes and the equivalent ball diameters (*d*_p_) were determined with the program ImageJ 1.52a. The temperature dependence of the relative permittivity of Fe_*x*_/(BaTiO_3_)_1−*x*_ ceramics (*x* = 0.1 to 0.6) in the range between 323 and 453 K was studied using a HP4192A Impedance Analyzer. A Quantum Design PPMS-9 was used for magnetic and magnetoelectric (ME) measurements. The ME measurements were performed with the magnetic field parallel and perpendicular to the electrical polarization. Before the magnetoelectric measurements, 100 nm thick gold electrodes were sputtered on both sides of the Fe/BaTiO_3_ composite ceramics, which had a height of 0.8–0.9 mm, with a Cressington Sputter Coater 108auto. Electrical poling was done with a Heinzinger LNC 1200-50 pos voltage supply at room temperature with a voltage of 800 V for 18 hours. Afterwards, the samples were short-circuited for 10 minutes. In the magnetoelectric measurements a small magnetic AC field (*H*_ac_) of 10 Oe, which was induced by an integrated solenoid, was superimposed to the static magnetic field. By measuring the in-phase voltage (*U*_ME_) *via* lock-in technique and considering the sample thickness (*d*) and the alternating magnetic field strength (*H*_ac_) the magnetoelectric coefficient (*α*_ME_) was calculated with eqn (1). The technical specifications of the experimental set-up has been described in ref. 38. The ME coefficient was studied at 300 K in a static magnetic field of −15 kOe ≤ *H*_dc_ ≤ 15 kOe with *f*_(Hac)_ = 900 Hz. The frequency dependence *α*_ME_(*f*_(Hac)_) was measured between 1 Hz up to 1000 Hz at 300 K and the temperature dependence of *α*_ME_ was analyzed between 300 and 10 K with *f*_(Hac)_ = 900 Hz. *α*_ME_(*f*_(Hac)_) and *α*_ME_(*T*) were measured at the dc field at which the maximum *α*_ME_ values were observed.1
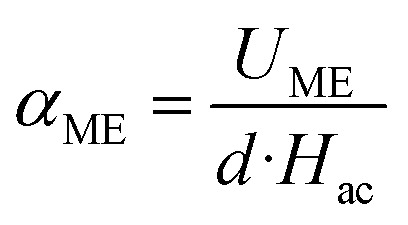


## 3. Results and discussion

### 3.1. Composition and morphology

**Fig. 1:**
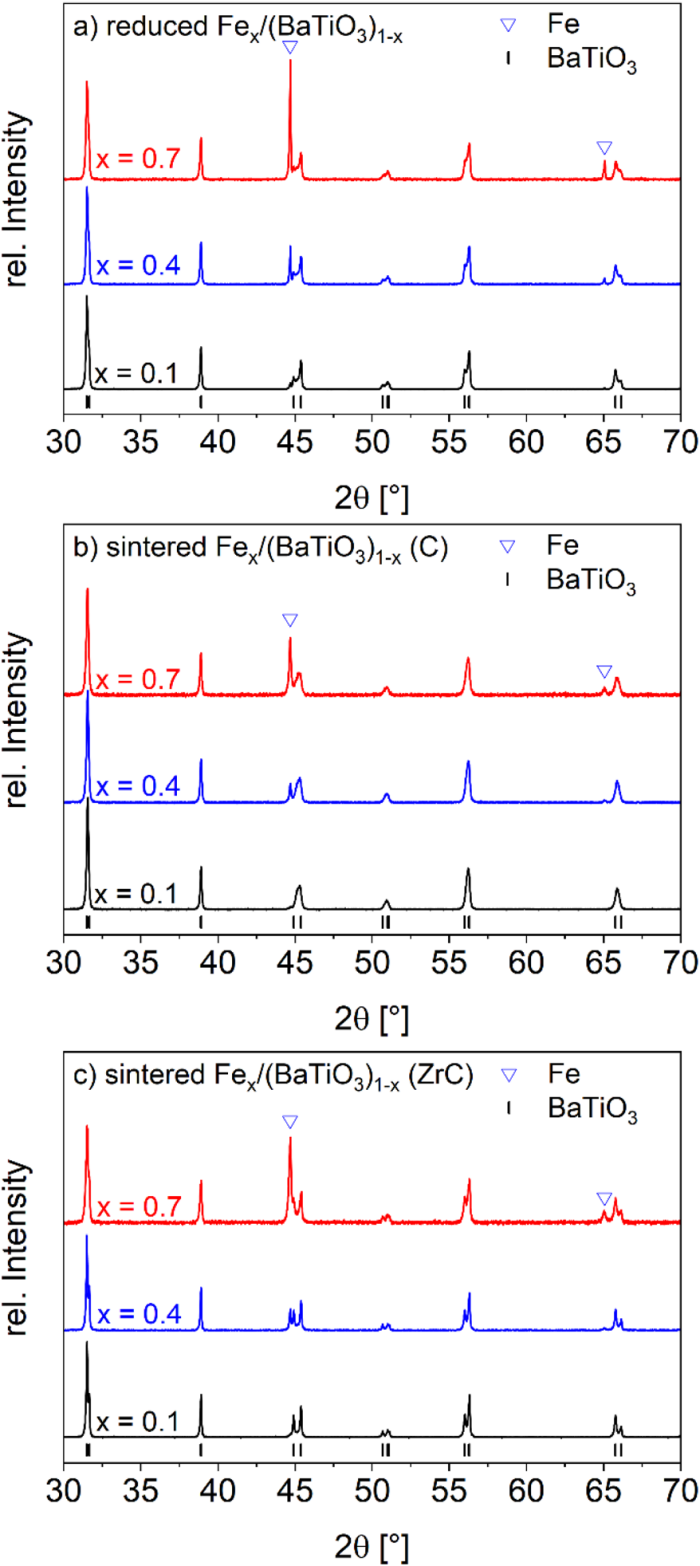
XRD pattern of selected Fe_*x*_/(BaTiO_3_)_1−*x*_ composites (*x* = 0.1, 0.4, 0.7) after reducing in forming gas at 1073 K for 2 h (a) and after sintering in nitrogen at 1623 K for 2 h with carbon (b) or zirconium carbide as oxygen getter (c).

It should be mentioned that the direct approach of sintering pellets of metallic iron and BaTiO_3_ was not successful because of bad pressing behavior and in turn unstable pellets. The first step in the synthesis of Fe/BaTiO_3_ composites was the reduction of Fe_2_O_3_ in forming gas. The XRD patterns of three selected samples after this step shown in Fig. 1a (XRDs of all samples are shown in the SI Fig. S1a) reveal the presence of tetragonal barium titanate and metallic iron. To get dense ceramics, an additional sintering step was carried out in inert atmosphere. In contrast to previous work on Ni and Co containing composites,^13,14^ the adjustment of a proper oxygen partial pressure was found to be more difficult. When very strong oxygen getters like titanium or forming gas are used conductive samples result because of the formation of high contents of oxygen defects in the BaTiO_3_ matrix. Such samples show no magnetoelectric response. On the other hand, a too high oxygen partial pressure can result in the reoxidation of Fe to iron oxide.

In case of Ni/(BaTiO_3_) or Co/BaTiO_3_ composites, the combination of nitrogen (5N purity) and carbon as oxygen getter was successfully used. For iron, on the other hand the Ellingham diagram^39^ based on the data of Barin^40^ (Fig. S2, SI) shows that the oxidation of CO to CO_2_ has a less negative Gibbs free energy than the oxidation of Fe to FeO at 1623 K. This means that carbon dioxide can oxidize metallic iron and in turn lead to the formation of iron-doped BaTiO_3_. In fact, the comparison of the XRD of selected samples after the sintering Fig. 1b with the ones after the first (reduction) step 1a (respectively Fig. S1a and b for all samples) reveals a less pronounced tetragonal splitting of the peaks as typically observed for iron-substituted BaTiO_3_.^25,41^ Therefore, other oxygen getters, namely Zr and ZrC, were investigated. Partial oxygen pressures of 10^−17^ bar (C), 10^−19^ bar (ZrC) and of 10^−21^ bar (Zr) were measured with a zirconia oxygen sensor at the furnace outlet. Promising composite samples were obtained with zirconium carbide (ZrC) and zirconium (Zr). Unfortunately, with zirconium as oxygen getter the composites become conductive and were therefore not further investigated. The powder diffraction pattern for the samples with *x* = 0.1, 0.4 and 0.7 after sintering with zirconium carbide are shown in Fig. 1c (all samples in Fig. S1c) and reveal the presence of pure iron and tetragonal barium titanate. Composites sintered with C as an oxygen getter exhibited an average crystallite size of ≈60 nm while for composites sintered in the presence of ZrC an average value of ≈74 nm was found but with a spread in the range of several tens of nanometers. The individual values of the crystallite sizes are shown in Fig. S2 in the SI and no clear trend with increasing iron content was observed.

For determination of the cell parameters Rietveld refinements were used (illustrated by the example Fe_0.4_/(BaTiO_3_)_0.6_ shown in Fig. S4). While the cell parameters of Fe were very similar in all samples with values of *a* = 2.865(2) Å (body-centered cubic iron), the obtained values for barium titanate in the samples after sintering with carbon (*a* = 4.001(1) Å and *c* = 4.020(1) Å) deviated significantly from the samples after sintering with zirconium carbide (*a* = 3.995(1) Å and *c* = 4.031(2) Å). In literature, cell parameters for iron (*a* = 2.8607 (2) Å), barium titanate (*a* = 3.995 Å and *c* = 4.034 Å) and iron doped barium titanate (BaFe_0.03_Ti_0.97_O_3_, *a* = 4.0006 Å and *c* = 4.0174 Å) have been reported and point to an iron doping of the barium titanate in the sintered composites with C as oxygen getter.^25,41,42^ In contrast, the cell parameters of the zirconium carbide sintered composites showed no indication for a doping of the BaTiO_3_ component.

**Fig. 2:**
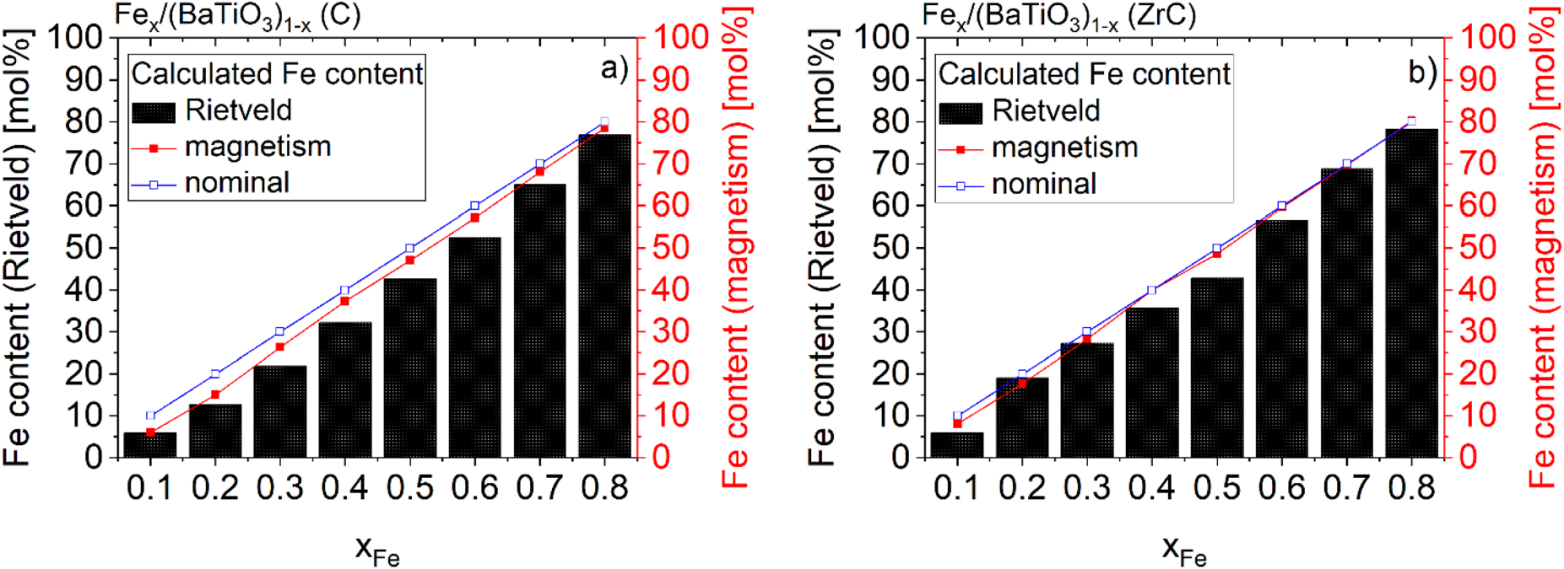
Comparison of the calculated Fe contents of Fe_*x*_/(BaTiO_3_)_1−*x*_ (*x* = 0.1–0.8) composites sintered with C (a) and ZrC (b) as oxygen getter based on Rietveld refinements and magnetic investigations (connecting lines are a guide to the eyes).

The contents of metallic iron were also determined by Rietveld refinements from the scaling factors of the two phases and are given in Table 1 and Fig. 2 together with the results of the magnetic investigations discussed below. The maximum deviations from the nominal iron content of 8.1 mol% was found for Fe_0.3_/(BaTiO_3_)_0.7_ sintered with C and 7.2 mol% in the case of Fe_0.5_/(BaTiO_3_)_0.5_ sintered with ZrC as oxygen getter. The minimum deviations in the range of 1.0 mol% was detected for Fe_0.2_/(BaTiO_3_)_0.8_ (ZrC). In general, the iron contents after sintering with zirconium carbide (ZrC) are in better agreement with the nominal values than the ones after sintering with carbon (C). This finding supports the interpretation of an iron doping of the barium titanate in the latter case.

**Table 1:** Saturation magnetization (*M*_S_), remanent magnetization (*M*_R_), iron content (calculated based on magnetic measurements and Rietveld refinements) and relative densities of Fe_*x*_/(BTO3)_1−*x*_ composites and pure iron (for comparison)

Nominal Fe content *x*	Oxygen getter	*M*_S_ [emu per g (Fe) (μ_B_ per f.u. Fe)]	*M*_R_ [emu per g (Fe) (μ_B_ per f.u. Fe)]	Calculated Fe content (magnetism) [mol%]	Calculated Fe content (Rietveld) [mol%]	Relative density [%]
0.1	C	130.5 (1.305)	0.072 (0.0007)	6.1	6	92.9
	ZrC	175.3 (1.753)	0.11 (0.0011)	8.1	6	97.9
0.2	C	158.5 (1.585)	0.15 (0.0015)	15.1	12.8	94.8
	ZrC	190.5 (1.905)	0.24 (0.0024)	17.7	19	98.7
0.3	C	185.6 (1.857)	0.32 (0.0032)	26.3	21.9	94.7
	ZrC	204.2 (2.042)	0.25 (0.0025)	28.3	27.3	96.1
0.4	C	199.2 (1.992)	0.13 (0.0013)	37.3	32.2	92.3
	ZrC	219.4 (2.194)	0.34 (0.0034)	40	35.6	96,9
0.5	C	200.0 (2.000)	0.11 (0.0011)	47.1	42.7	92.0
	ZrC	210.1 (2.101)	0.32 (0.0032)	48.7	42.8	97.0
0.6	C	201.4 (2.104)	0.08 (0.0008)	57.2	52.5	93.8
	ZrC	217.7 (2.177)	0.32 (0.0032)	59.7	56.6	97.1
0.7	C	207.2 (2.072)	0.08 (0.0008)	68.1	65.1	90.9
	ZrC	218.0 (2.180)	0.57 (0.0057)	69.8	68.8	97.6
0.8	C	209.9 (2.099)	0.14 (0.0014)	78.6	76.9	91.9
	ZrC	221.9 (2.219)	0.31 (0.0031)	80.3	78.3	96.7
Pure Fe		219.5 (2.195)	0.35 (0.0035)	100	100	76.6

Both series of composites exhibited densities above 90% of the crystallographic ones. These densities were calculated according to ref. 13, 14, 43 and are listed in Table 1. The densities of the carbon-sintered composites (*ca.* 91–95%) are slightly lower than the ones of the ZrC sintered ones (*ca.* 96–99%), which is assumed to result in a better magnetoelectric coupling for the samples sintered with zirconium carbide as oxygen getter.

**Fig. 3:**
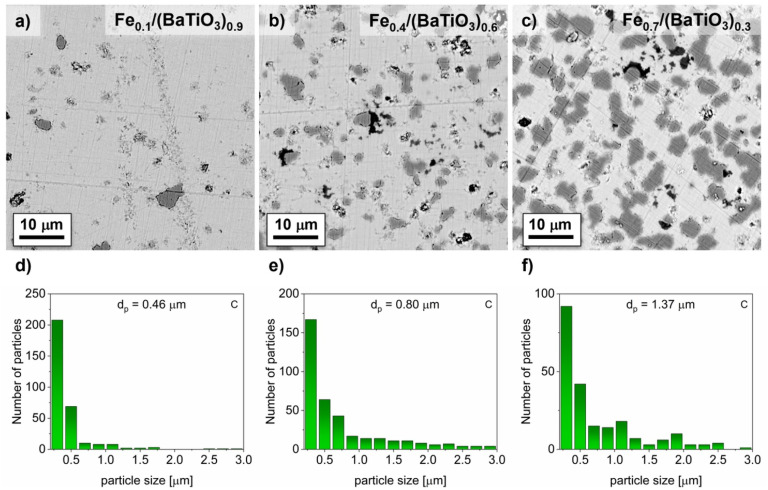
SEM images in back-scattered electron mode and distribution of average Fe particle size for selected Fe_*x*_/(BaTiO_3_)_1−*x*_ composites (*x* = 0.1 (a and d), 0.4 (b and e), 0.7 (c and f)) with C as oxygen getter with the equivalent ball diameters *d*_p_.

**Fig. 4:**
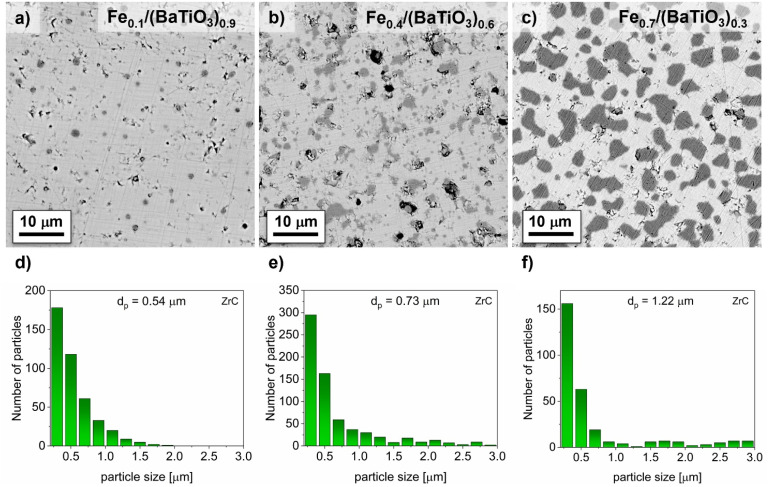
SEM images in back-scattered electron mode and distribution of average Fe particle size for selected Fe_*x*_/(BaTiO_3_)_1−*x*_ composites (*x* = 0.1 (a and d), 0.4 (b and e), 0.7 (c and f)) with ZrC as oxygen getter with the equivalent ball diameters *d*_p_.

The expected 0-3 connectivity of the composites was verified by SEM as shown in Fig. 3a–c and 4a–c for selected values of *x*.

All samples contained predominantly globular Fe particles embedded in the BaTiO_3_ matrix. The particle shapes varied from small and well isolated ones to larger and partly agglomerated Fe particles with increasing iron content. Assuming globular particles, the equivalent ball diameters can be calculated. In Fig. 3d–f and 4d–f the distributions and average values of selected Fe_*x*_/(BaTiO_3_)_1−*x*_ samples after sintering with the two different oxygen getters are given. Similar particle size distributions and a roughly linear increase of the equivalent ball diameter from 0.46 μm (for *x* = 0.1), 0.80 μm (for *x* = 0.4) to 1.37 μm (for *x* = 0.7) for carbon and from 0.54 μm (for *x* = 0.1), 0.73 μm (for *x* = 0.4) to 1.22 μm (for *x* = 0.7) for ZrC as oxygen getter were determined. These values are significantly larger than the crystallite sizes mentioned above. This indicates that each iron particle consists of several crystallites. Considering the minor differences in the particle sizes for samples with the same nominal compositions, no morphological differences were detected. Samples, which were sintered with carbon were found to be conductive (*ρ* < 20 GΩ) for *x* ≥ 0.6 whereas samples sintered with zirconium carbide showed significant electrical conductivities only for *x* ≥ 0.7.

### 3.2. Impedance and magnetic measurements

**Fig. 5:**
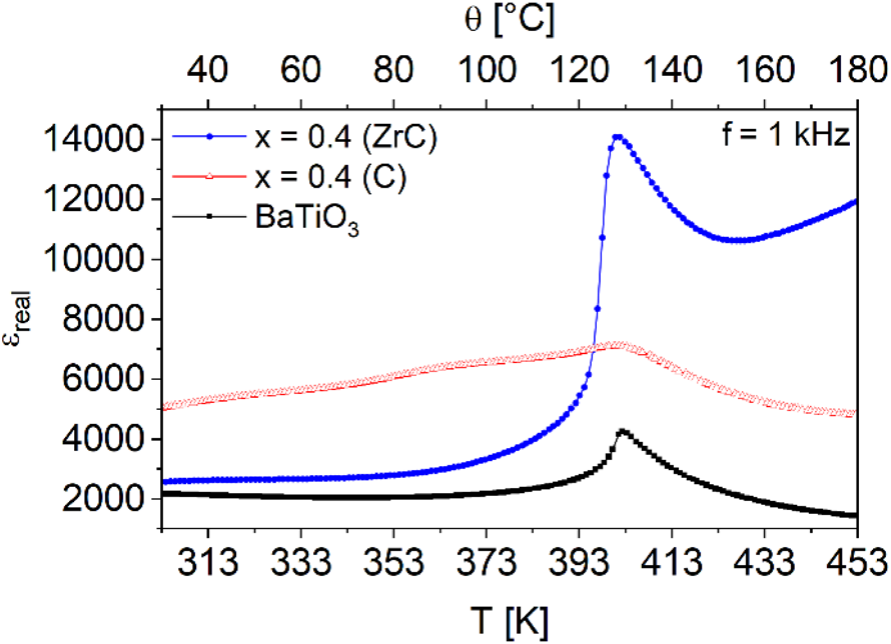
Impedance measurements of Fe_0.4_/(BaTiO_3_)_0.6_ composites sintered with C (red) and ZrC (blue) as oxygen getter in comparison to pure barium titanate (black).

**Fig. 6:**
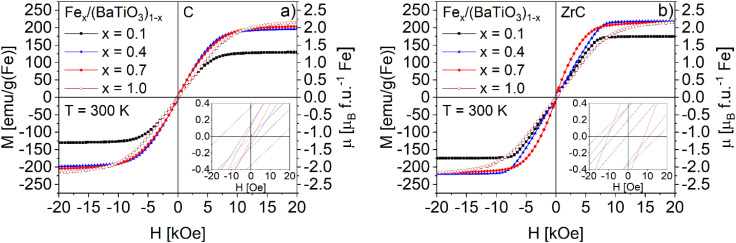
Field dependence of the magnetization of Fe_*x*_/(BaTiO_3_)_1−*x*_ composites (*x* = 0.1, 0.4 and 0.7) sintered with C (a) and ZrC (b) as oxygen getter (normalized to the nominal Fe content) and pure Fe. The insets show the low-field region.

The dielectric properties of the Fe_*x*_/(BaTiO_3_)_1−*x*_ composites were investigated by impedance spectroscopy. Fig. 5 shows the relative permittivities of selected composite samples Fe_0.4_/(BaTiO_3_)_0.6_ sintered with ZrC and C as oxygen getter in comparison to pure BaTiO_3_. The results of further impedance measurements are shown in Fig. S5 in the SI. The ferroelectric transition temperature of barium titanate from the cubic high temperature modification to the tetragonal one at *ca.* 395 K was observed in all three samples. For pure barium titanate and the composites sintered under the presence of ZrC very sharp transitions are found. In contrast, composite sintered with C showed a strong broadening of the transition peaks. In addition, a further broad and small peak can be seen at *ca.* 373 K. Pandey *et al.*^25^ investigated iron doping in barium titanate and observed a broadening of the ferroelectric phase transition and a broad diffuse peak at around 370 K in their impedance measurements, too. Based on these findings, a small iron doping for the carbon sintered composites can be deduced. The magnetic measurements at 300 K of both series of composites (with C and ZrC as oxygen getter) showed a slightly different magnetic behavior compared to pure iron prepared under the same conditions, as can be seen for selected Fe_*x*_/(BaTiO3)_1−*x*_ composites (with *x* = 0.1, 0.4 and 0.7) in Fig. 6a (C) and Fig. 6b (ZrC) and for all samples in Fig. S6a and b. The magnetization values have been normalized with respect to the nominal Fe content (*x*).

For both series of composites, the samples with the smallest iron content (*x* = 0.1) exhibit a reduced saturation magnetization. This effect is more pronounced for the sample sintered in the presence of carbon. These observations indicate a slight iron doping of the BaTiO_3_ matrix as discussed above. The influence of this doping on the magnetism is most visible for the lowest *x* values as the relative difference between relative and nominal Fe content is the largest for these samples. In case of the carbon sintered composites the magnetic behavior comes very close to the magnetic behavior of pure iron with increasing iron content but does not exactly reach its saturation magnetization.

On the other hand, the zirconium carbide sintered samples achieved the saturation magnetization of pure Fe for higher iron contents, but the saturation is reached at smaller magnetic fields. Changes in the Fe particle sizes seem unlikely as an explanation for the observed differences because of similar increasing particle size distributions and equivalent ball diameters of iron for both series of samples. A possible reason might be the formation of a heavily iron doped BaTiO_3_ layer in the case of composites sintered with carbon. The calcination step at 1623 K leads to a contraction of the BaTiO_3_ matrix, putting the iron particles under compressive stress. The possible formation of an iron doped barium titanate layer at the interface of the two phases can act as a buffer and reduce the compressive stress and in turn affect the magnetic behavior as well as the magnetostriction.

In Table 1 the saturation magnetizations (*M*_S_) and remanent magnetizations (*M*_R_) of the Fe_*x*_/(BaTiO_3_)_1−*x*_ composites and pure iron are summarized.

Coercivities below 10 Oe cannot accurately be determined with the PPMS because of an intrinsic remanence (up to 20 Oe) of the superconducting magnet and are therefore not listed.^44^

Using the saturation magnetization of 2.195*μ*_B_ for pure iron (*x* = 1) the iron contents of the composites can be calculated from their *M*_S_ values. The results for the two series of samples are listed in Table 1 and are shown in Fig. 2. The composites sintered in the present of carbon showed deviations from 1.4 (*x* = 0.8) up to 4.9 mol% (*x* = 0.2). In comparison, the deviations from the nominal iron contents of the composites sintered with zirconium carbide are lower and range from 0.3 mol% (*x* = 0.4, 0.6, 0.7 and 0.8) to 2.3 mol% (*x* = 0.2). These findings are in accordance with the results of the Rietveld refinements discussed above (see Fig. 2) and show that ZrC prevents the incorporation of iron in BaTiO_3_.

### 3.3. Magnetoelectric measurements

**Fig. 7:**
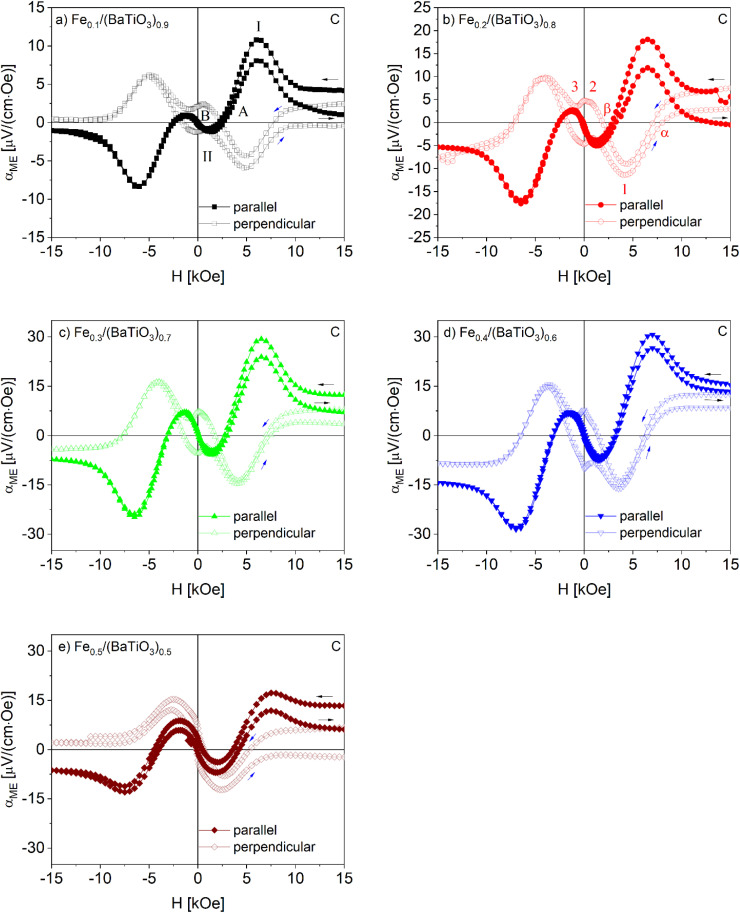
Field dependence of the magnetoelectric coefficient of Fe_*x*_/(BaTiO_3_)_1−*x*_ samples (*x* = 0.1 (a), 0.2 (b), 0.3 (c), 0.4 (d) and 0.5 (e)) sintered with C as oxygen getter in parallel and perpendicular orientation. Numbers and letters at special positions are described in the text.

**Fig. 8:**
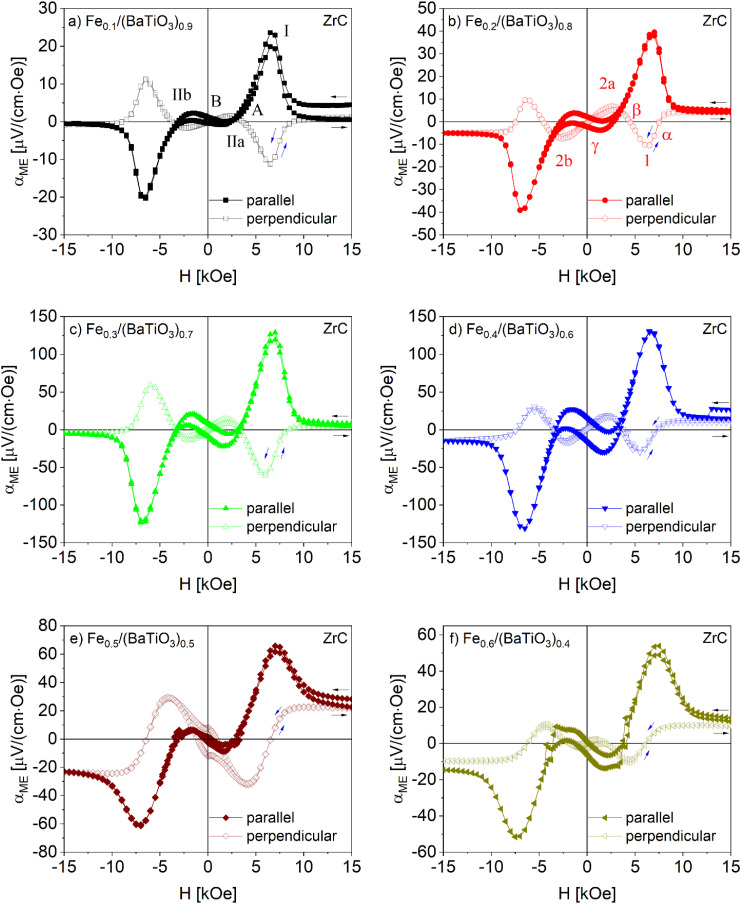
Field dependence of the magnetoelectric coefficient of Fe_*x*_/(BaTiO_3_)_1−*x*_ samples (*x* = 0.1 (a), 0.2 (b), 0.3 (c), 0.4 (d), 0.5 (e) and 0.6 (f)) sintered with ZrC as oxygen getter in parallel and perpendicular orientation. Numbers and letters at special positions are described in the text.

Magnetoelectric measurements were performed at 300 K in the field range ±15 kOe for iron contents up to 50 mol% (C) respectively 60 mol% (ZrC). For higher Fe contents the samples were too conductive to be investigated. Both for the parallel and perpendicular orientation of polarization and magnetic field a complex field dependence with several characteristic points was observed as shown in Fig. 7 and 8.

We start the discussion of *α*_ME_ at +15 kOe. For the collinear orientation (*H*‖*P*) the magnetoelectric coefficient starts at comparatively small values, passes a first maximum (marked as I) at a field of roughly 7.5 kOe denoted as *H*_I_ (Fig. 7a and 8a) followed by a first coercive field (*H*_C_) at the point A and a minimum (II respectively IIa) at *H*_II_/*H*_IIa_ of about 2.5 kOe. Near 1500 Oe a second zero crossing of the magnetic field axis occurred (B).

An interesting difference was found at *H* = 0 for the samples sintered with C and ZrC respectively. While for the former *α*_ME_ is zero, samples sintered with ZrC showed a hysteresis with a more or less pronounced remanence (*α*_ME,rem_) at zero field. For ‘negative’ magnetic fields (*i.e.* opposite direction of the field) an inverted behavior of *α*_ME_ was observed: Upon increasing the field, a maximum at roughly −2.5 kOe occurs followed by a minimum near 7.5 kOe and finally a small positive or negative *α*_ME_ value at the maximum negative field (−15 kOe).

When the magnetic field is switched back from −15 kOe to +15 kOe, *α*_ME_ follows the same course for the samples sintered with carbon as oxygen getter but does not reach the original values at the maximum field of 15 kOe. This most likely results from a loss of polarization due to leaky currents in the samples. In contrast, the samples sintered with ZrC showed the mentioned hysteresis at low fields and additionally nearly the same ME coefficients are found after the full field sweep (with the exception of *x* = 0.1), reflecting a less defective nature of the samples.

In case of the magnetoelectric investigations in the perpendicular sample set-up (*H*⊥*P*) in general an inverted behavior is found, *i.e.* a maximum for *H*‖*P* corresponds to a minimum for *H*⊥*P* and *vice versa*. Still significant differences were observed with respect to the magnetic fields at which the extrema are found and the remanences. Upon decreasing the magnetic field from +15 kOe the samples sintered with carbon as oxygen getter showed a minimum (marked as 1 in Fig. 7b) at a magnetic field *H*_I_ around 5 kOe, *i.e.* at a field which is much smaller than *H*_I_ (maximum of *α*_ME_ for *H*‖*P*). While further decreasing the magnetic field, a small maximum (point 2) near zero field occurs and at zero field a significant remanent ME coefficient (*α*_ME,rem_) remains. When the field is enhanced to −15 kOe an inverted behavior of *α*_ME_ is observed and (as in the case of *H*‖*P*) the starting values are not reached when the field is again raised to +15 kOe. A remarkable difference was found for *x* = 0.5, for which no remanent magnetoelectric coefficient appears.

Also for the samples sintered in the presence of ZrC as oxygen getter a principally inverted behavior of the *α*_ME_ courses for *H*‖*P* and *H*⊥*P* was found. On the other hand, the characteristic fields at which the maxima/minima occur are more similar than for the C sintered samples. It is to be noted, though, that the field differences increase with larger *x* values as can be seen when comparing *e.g.*Fig. 8a and d. For the series of composites sintered in the presence of ZrC, remanent *α*_ME_ values occur for *H*‖*P* while they are missing for *H*⊥*P*. This is the opposite behavior than found for the C sintered samples.

As shown in Fig. 8e and f, a somewhat deviating trend was observed for the composites with highest iron contents (*x* = 0.5 and 0.6).

In Tables 2 and 3 a compilation of the measured values of *α*_ME_ and the corresponding magnetic fields at the above-specified points is given. For convenience only the averaged absolute values are listed. The field-dependent magnetoelectric investigations of all samples are additionally shown in Fig. S7a and b for samples sintered with C and Fig. S8a and b with ZrC in a different way to ease comparison.

**Table 2:** Magnetoelectric properties (*H*_C,ME_ = magnetoelectric coercivity, *H*_max/min_ = magnetic field of the maximum/minimum of *α*_ME_, *α*_ME,ext_ = maximum/minimum values of *α*_ME_ at *H*_max_) of Fe_*x*_/(BaTiO_3_)_1−*x*_ composites measured in the parallel sample setup at specific points (see Fig. 7a und 8a)

	Fe content *x*		0	0.1	0.2	0.3	0.4	0.5	0.6
C	*H*_C,ME_ [Oe]	A	—	2428	2816	2989	3190	4065	—
		B	—	102	79	43	118	289	—
	*H*_max/min_ [Oe]	I	—	6500	6500	6500	7000	7500	—
		II	—	1300	1200	1400	1600	1800	—
	*α*_ME,ext_ [μV cm^−1^ Oe^−1^]	I	—	9	16	26	29	13	—
		II	—	−1	−4	−6	−7	−7	—
ZrC	*H*_C,ME_ [Oe]	A	—	2750	2751	3225	3075	3207	3619
		B	—	535	—	1041	1587	384	744
	*H*_max/min_ [Oe]	I	—	6500	7000	7000	6500	7000	7500
		IIa	—	1600	1300	1600	1600	1600	1800
		IIb	—	1800	1500	2250	2250	—	2250
	*α*_ME,ext_ [μV cm^−1^ Oe^−1^]	I	—	21	39	123	131	62	52
		IIa	—	−2	−4	−22	−29	−7	−11
		IIb	—	1	0.5	6	2	—	4

**Table 3:** Magnetoelectric properties (*H*_C,ME_ = magnetoelectric coercivity, *H*_max/min_ = magnetic field of the maximum/minimum of *α*_ME_, *α*_ME,ext_ = maximum/minimum values of *α*_ME_ at *H*_max_) of Fe_*x*_/(BaTiO_3_)_1−*x*_ composites measured in the perpendicularl sample setup at specific points (see Fig. 7a und 8a)

	Fe content *x*		0	0.1	0.2	0.3	0.4	0.5	0.6
C	*H*_C,ME_ [Oe]	α	—	—	7411	7489	6420	—	—
		β	—	1990	1818	1601	1420	407	—
	*H*_max/min_ [Oe]	1	—	5000	4000	4000	3750	2500	—
		2	—	300	0	0	0	—	—
		3	—	700	800	800	900	—	—
	*α*_ME,ext_ [μV cm^−1^ Oe^−1^]	1	—	−6	−10	−15	−15	−12	—
		2	—	1	5	6	9	—	—
		3	—	1	3	4	7	—	—
ZrC	*H*_C,ME_ [Oe]	α	—	8947	7509	8479	7346	6479	6304
		β	—	3653	4519	3337	3595	988	1562
		γ	—	316	20	302	65	—	—
	*H*_max/min_ [Oe]	1	—	6500	6500	6000	5250	4250	4250
		2a	—	2500	2500	2000	2000	0	700
		2b	—	2250	2750	2250	—	—	1300
	*α*_ME,ext_ [μV cm^−1^ Oe^−1^]	1	—	−11	−10	−60	−29	−31	−7
		2a	—	2	7	15	17	10	2
		2b	—	1	6	9	—	—	0.05

Composites with an iron content of 40 mol% showed the highest magnetoelectric coefficient of *α*_ME,ext_ = 29 μV cm^−1^ Oe^−1^ (C) and *α*_ME,ext_ = 131 μV cm^−1^ Oe^−1^ (ZrC) in the parallel sample setup (*H*‖*P*). In contrast, the magnetoelectric investigations in the perpendicular arrangement (*H*⊥*P*) exhibited the highest values of *α*_ME,ext_ = −15 μV cm^−1^ Oe^−1^ (C) and *α*_ME,ext_ = −60 μV cm^−1^ Oe^−1^ (ZrC) for samples with *x* = 0.3. These results are in line with the investigations of the Ni_*x*_/(BaTiO_3_)_1−*x*_ and Co_*x*_/(BaTiO_3_)_1−*x*_ composites.^13,14^

Comparing the two different composite synthesis conditions, the sintering with ZrC as oxygen getter results in higher values of *α*_ME,ext_ than for C as oxygen getter at same iron contents. This might be a consequence of the iron doping of the barium titanate in the second series of samples for several reasons.

First, the reduction of the amount of iron leads to a weaker magnetostriction, which directly influenced the magnetoelectric effect because of the mechanical coupling to the piezoelectric component *α*_ME_ ∼ (d*E*/d*S*)(d*S*/d*H*).^4,13,14,45,46^ Second, the possible formation of an interlayer between the magnetostrictive and piezoelectric phase can reduce the mechanical propagation because the interlayer most likely lacks (at least) one or both ferroic properties. Finally, the densities of the composites sintered with ZrC are slightly higher, which points to a more intimate connection of the two components.

**Fig. 9:**
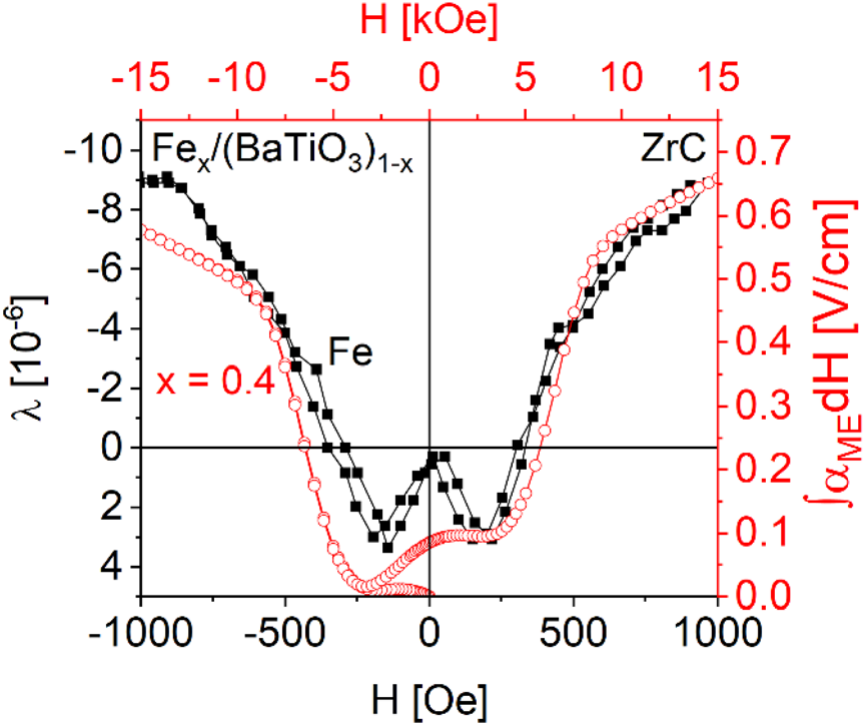
Comparison of the magnetostriction of iron (using data of Chen *et al.*^19^) and the integration of *α*_ME_ of the Fe_0.4_/(BaTiO_3_)_0.6_ composite sintered with ZrC as oxygen getter.

In our investigations of the magnetoelectric effect a small AC driving field of 10 Oe is superimposed parallel to the DC bias field. Accordingly, *α*_ME_ is expected to reflect the slope of the magnetostriction d*λ*/d*H* or, in other words, the integral ∫*α*_ME_d*H* and *λ* of iron should show the same field dependence. Fig. 9 shows a comparison of the integral of *α*_ME_ for Fe_0.4_/(BaTiO_3_)_0.6_ in parallel orientation (*H*‖*P*) and the magnetostrictive coefficient *λ* of pure iron, for which the data was taken from ref. 19 (the integrals of *α*_ME_ for all Fe_*x*_/(BaTiO_3_)_(1−*x*)_ samples in parallel orientation are depicted in Fig. S9). As can be seen in Fig. 9, both courses show a similar behavior. On the other hand, the magnetic fields differ by almost one order of magnitude (please mind the two different *x*-scales). Similar findings were already observed for Co_1−*x*_Ni_*x*_Fe_2_O_4_/BaTiO_3_ and Ni_*x*_/(BaTiO_3_)_(1−*x*)_.^6,13^

The field dependence of the derived magnetostriction of iron is quite unusual, starting with slightly positive values and becoming negative as the magnetic field increases. This is not the case for other magnetostrictive substances like nickel but has already been observed in mixtures of hexagonal and cubic cobalt. A prerequisite to observe the ME effect are highly dense ceramics with an intimate connection of the two components and a highly insulation ferroelectric matrix, which requires very carefully adjusted sintering conditions. The differences in the field dependence of the magnetostriction of pure iron and the ME signal of our composites might result from a confinement of magnetostriction of Fe due to the embedment in the BaTiO_3_ matrix. A conclusive description of the magnetoelectric behavior is difficult for a number of reasons, for example the distribution of particle/grain sizes of both components and the tensor characteristic of the ferroic properties^46^ in combination with the missing crystallographic relationships between the two components (*i.e.* statistical orientation of the various crystallographic domains).

Furthermore, the nature of the applied magnetic field plays an important role: Hristoforou *et al.*^47^ investigated *λ*(*H*) of an as-cast amorphous Fe_78_Si_7_B_15_ ribbon using DC and AC bias fields.

The measurements in a DC bias field exhibit a sharp turning point at zero field and the saturation magnetostriction is reached at a small magnetic field *ca.* 1000 Oe. In contrast, in the case of an AC bias field, a broad turning point at zero field is found and the saturation magnetostriction is reached at a much higher magnetic field of *ca.* 2000 Oe.

Despite of these obstacles Filippov *et al.*^48,49^ worked out a relationship between the magnetostrictive and magnetoelectric behavior of a multiferroic composite *via* an effective parameters method and obtained the following eqn (2):2
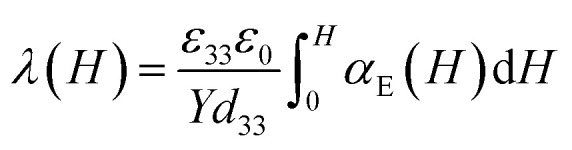
where *ε*_33_ is the z-component (parallel to the electric field) of the (relative) permittivity of the composite, *ε*_0_ is the vacuum permittivity, *Y* the Young's modulus and *d*_33_ the z-component of the piezoelectric tensor. Using this approach a description of the magnetostrictive behavior of NiFe_1.9_Co_0.02_O_4_/PZT850 composites with different ferrite content was carried out. Furthermore Filippov *et al.*^48^ proposed a description of the correlation of the magnetostrictive and magnetoelectric behavior of a multiferroic composite *via* the saturation magnetostriction of the composites:3
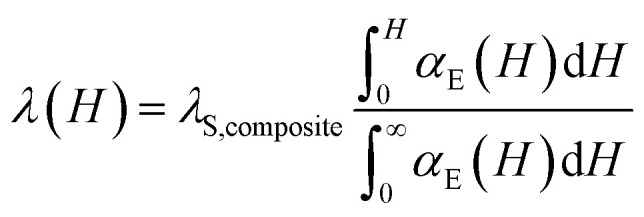


Following this model the saturation magnetostriction of our composite can be approximated based only on the fill fraction (*f*) and the Poisson's ratio of barium titanate (*σ*_BaTiO_3__):4
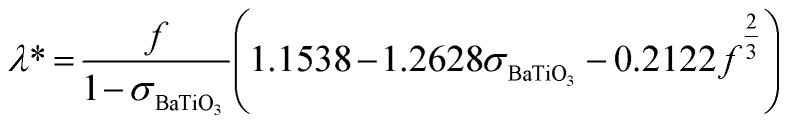
And5
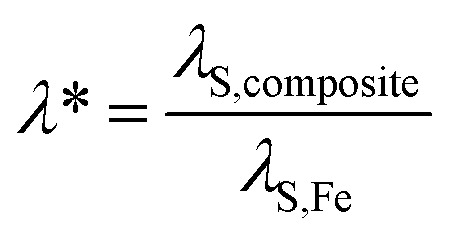


**Fig. 10:**
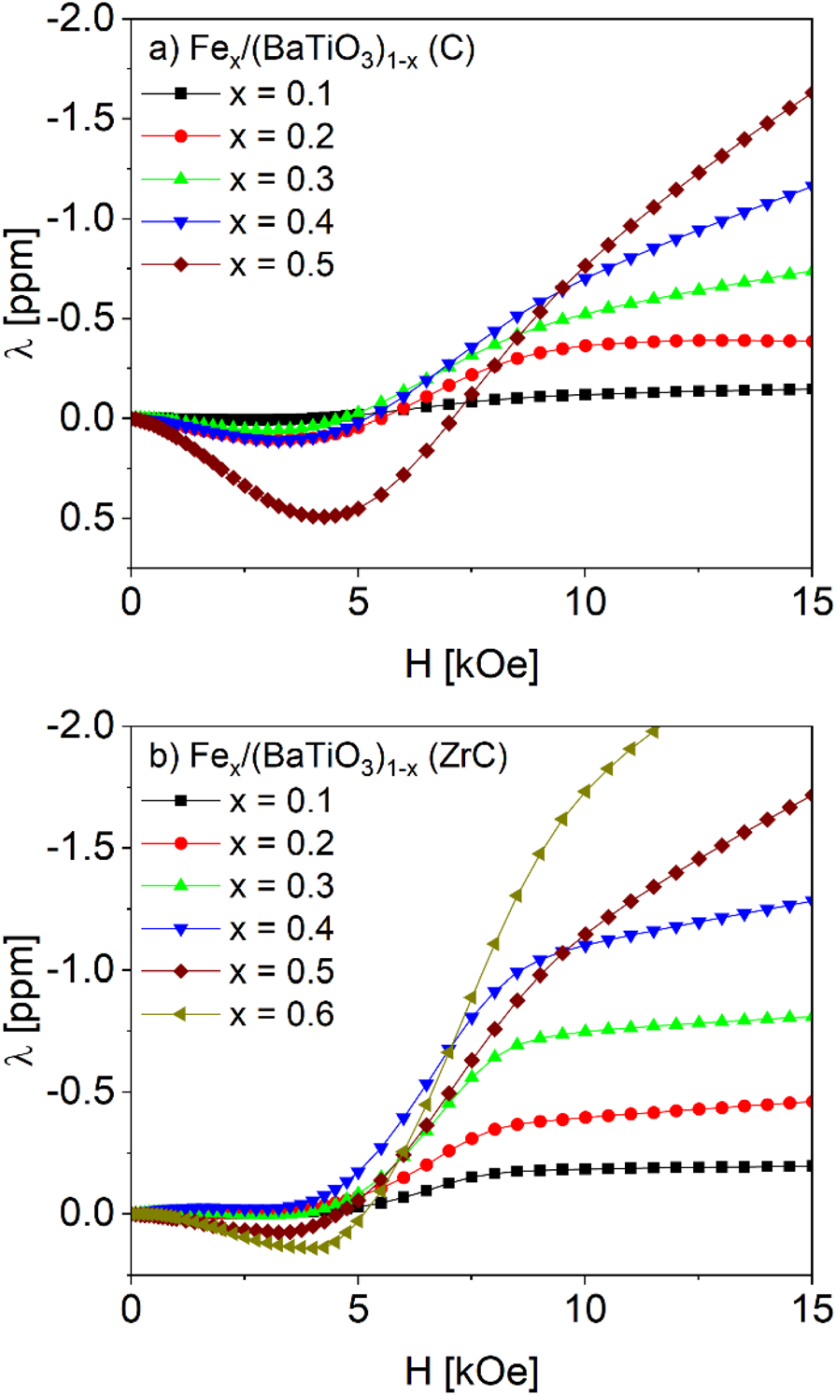
Theoretical magnetostrictive behavior of Fe_*x*_/(BaTiO_3_)_1−*x*_ composites (*x* = 0.1 up to 0.5 respectively 0.6) with C (a) and ZrC (b) as oxygen getter calculated after ref. 48 and 50.

Using the Poisson ratio *σ*_BaTiO_3__ = 0.33,^51^ the saturation magnetostriction of pure iron *λ*_S,Fe_ = −10 ppm (ref. 19) and the integrations of the measured magnetoelectric values at 0 ≤ *H* ≤ 15 kOe and ∞ (*H* = 15 kOe) the magnetostrictive behavior of Fe_*x*_/(BaTiO_3_)_1−*x*_ composites can be calculated *via*eqn (3)–(5). The results are shown in Fig. 10. The comparison of ∫*α*_ME_d*H* (see Fig. 9) and the calculated values of *λ* for the composites shows a similar behavior: Starting with small values for *H* = 0 a maximum is reached at roughly 2.5–5 kOe, followed by a zero-crossing and (more or less) saturation at 15 kOe. Thus, the method of Filippov *et al.*^48^ provides a suitable way for describing the magnetoelectric behavior of our composites.

The frequency dependence of the magnetoelectric coefficient was measured at 300 K at the magnetic field *H*_max_ (*i.e.* about 7 kOe). Similar to previous studies^13,14^ an increase up to 400 Hz was found for both orientations (*H*‖*P* and *H*⊥*P*). Between 400 and 1000 Hz the magnetoelectric effect is nearly field-independent. Higher frequencies cannot be measured with our set-up.

Results for selected Fe_*x*_/(BaTiO_3_)_1−*x*_ composites sintered with ZrC as oxygen getter are shown in Fig. S10.

**Fig. 11:**
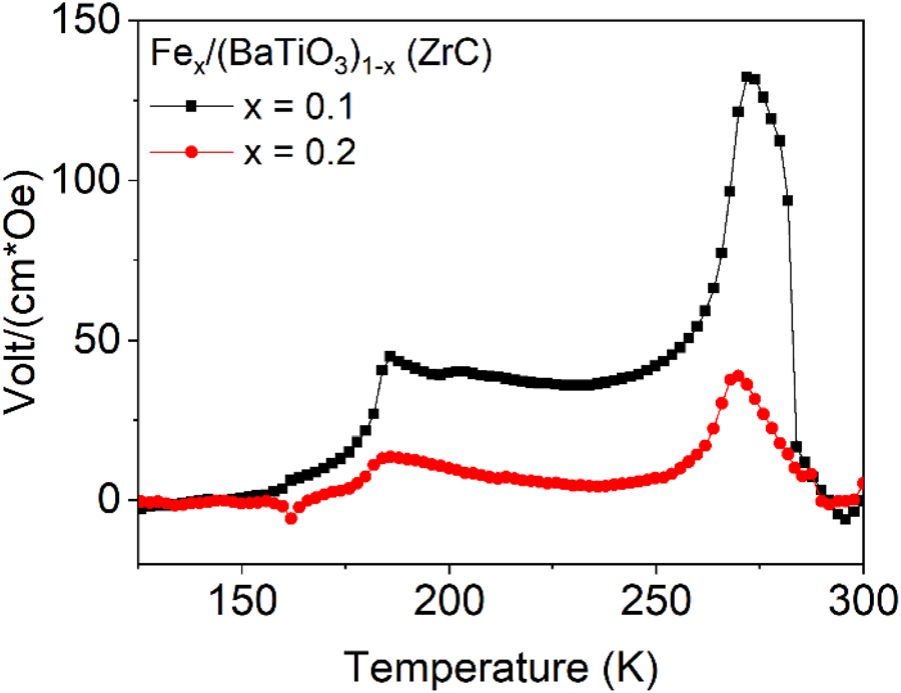
Temperature dependent magnetoelectric coefficient in the parallel sample setup of the Fe_0.1_/(BaTiO_3_)_0.9_ and Fe_0.2_/(BaTiO_3_)_0.8_ composites sintered with ZrC as oxygen getter.

The temperature dependence of *α*_ME_ for the two samples Fe_*x*_/(BaTiO_3_)_1−*x*_ with *x* = 0.1 and 0.2 using ZrC as oxygen getter are shown for the parallel sample setup (*H*‖*P*) in Fig. 11. A background has been modelled with a Lorenz function as described in ref. 14 and subtracted. Two characteristic features can be seen at 270 and 184 K. These temperatures are in good agreement with literature values for the tetragonal to orthorhombic (269 K) and the orthorhombic to rhombohedral phase transition temperature (177 K) of BaTiO_3_.^12^

This proves that the phase transitions of the ferroelectric component directly affect *α*_ME_ and this method can be used to characterize the phase transitions of barium titanate.

**Fig. 12:**
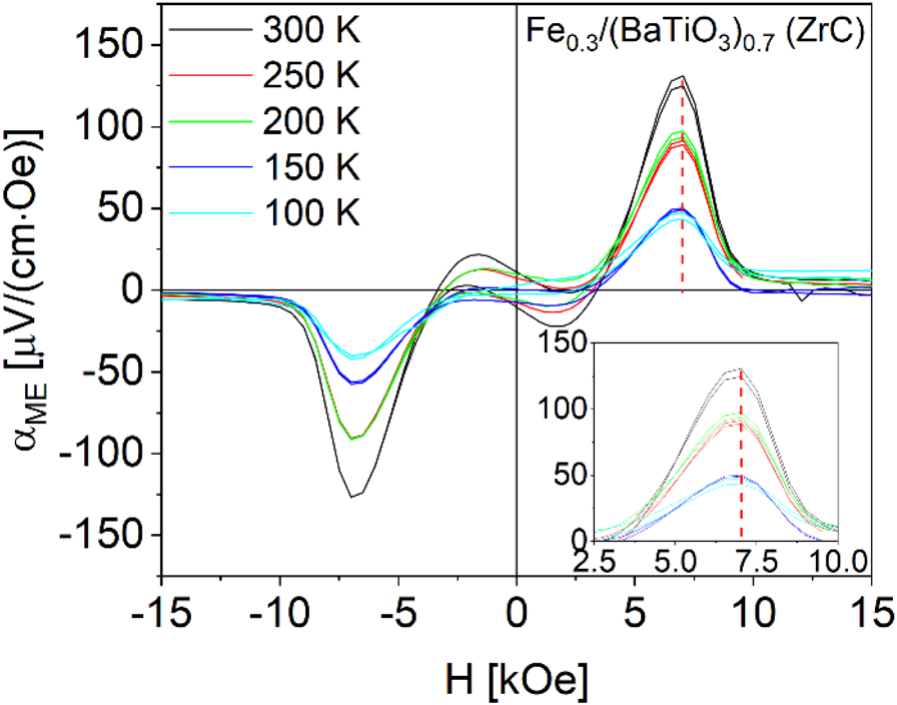
Magnetoelectric measurements at different temperatures in the parallel sample setup of Fe_0.3_/(BaTiO_3_)_0.7_ sintered with ZrC as oxygen getter.

Additional DC field dependent investigations of the magnetoelectric coefficient were performed at different temperatures (*T* = 300, 250, 200, 150 and 100 K) for Fe_0.3_/(BaTiO_3_)_0.7_ are shown in Fig. 12. No temperature shift of the positions of the *α*_ME_ maxima/minima was detected. The values at 300 K are highest and measurements at 250 and 200 K as well as 150 and 100 K show similar behaviors and values.

This correlates with the phase transition temperatures of barium titanate, because at 300 K BaTiO_3_ exists in the tetragonal phase, while at 250 and 200 K the orthorhombic and between 150 and 200 K the rhombohedral phase is stable. The cubic to tetragonal transition of barium titanate at 493 K cannot be investigated in our set-up as its temperature is limited to 350 K.

In summary, the magnetoelectric measurements allow the characterization of the magnetostrictive respectively piezoelectric properties of a composite and phase transitions can be detected as well.

## 4. Conclusion

Mixtures of Fe_2_O_3_ and BaTiO_3_ can be reduced in forming gas and subsequently sintered in nitrogen with carbon respectively zirconium carbide as oxygen getter to form Fe_*x*_/(BaTiO_3_)_1−*x*_ composites (*x* = 0.1–0.8) with 0-3 connectivity and densities above 90% (C) respectively above 95% (ZrC) of the crystallographic ones. The samples were insulating up to an iron content of *x* = 0.5 (C) and *x* = 0.6 (ZrC). XRD Rietveld analyses showed a strong shift of the cell parameters of barium titanate towards a cubic cell metric in the case of C whereas composites sintered with ZrC exhibited the cell parameters of tetragonal barium titanate. The shift of cell parameters after sintering in the presence of carbon points to an iron doping of barium titanate as additionally supported by temperature-dependent impedance measurements. The 0-3 connectivity was examined by SEM showing well embedded, rather globular iron particles in the surrounding barium titanate matrix. For both composite series, the particle sizes (equivalent ball diameters) increase with rising iron content (*x*). The composites sintered in the presence of carbon showed significant discrepancies between the nominal and the experimental iron contents as determined by magnetic investigations and XRD-Rietveld refinements, whereas sintering with ZrC led to values close to the expected ones.

Magnetoelectric investigations were carried out depending on magnetic field, frequency of the driving ac-field and temperature and a complex magnetoelectric behavior was found. All samples showed a centrosymmetric *α*_ME_*vs. H* behavior in the perpendicular and parallel sample set-up (*H*‖*P*, *H*⊥*P*) but different behavior and values of the magnetoelectric coefficient depending on the synthesis and orientation. Generally, a minimum at *ca.* 1600 Oe and a maximum at *ca.* 7000 Oe are found in the parallel set-up for both sample series. The maximum *α*_ME_ values were in the range 10–30 μV cm^−1^ Oe^−1^ for samples sintered in the presence of carbon for *H*‖*P*. In perpendicular set-up (*H*⊥*P*) in principle a mirrored behavior with smaller ME coefficients were found and a remanence at *H* = 0 was observed. The ZrC sintered composites exhibited significantly higher magnetoelectric coefficients up to 130 μV cm^−1^ Oe^−1^. The low hysteresis in the range ± 5000 Oe and the remanent magnetoelectric coefficient was found for *H*‖*P* but missing for *H*⊥*P*. The complex *α*_ME_ behavior can be related to the magnetostrictive behavior of iron using the theorical approach of Filippov *et al.*^48^

The magnetoelectric coefficient increases with the frequency of the driving ac-field up to 400 Hz and remains basically constant up to 1000 Hz. Temperature dependent measurements showed a maximum at *ca.* 270 K and a step-like structure at *ca.* 175 K, correlating with the phase transition temperatures of BaTiO_3_ and reflecting the deviating ferroelectric properties of the different crystallographic phases.

Our investigations show that magnetoelectric composites consisting of iron and barium titanate can be obtained in a convenient way by reducing a mixture of Fe_2_O_3_ and BaTiO_3_. To achieve a high density and in turn a close connection of the two components, a sintering step in inert atmosphere had to be applied. It turned out that the physical properties of the obtained composites strongly depend on the oxygen getter used in the sintering step. ZrC gave much better results in most respects compared to carbon. Our study helps to improve the understanding of the magnetoelectric effect of composites and supports their application. Due to the significant remanent ME signal and the fact that even small changes of the magnetic field can cause an extinction or a reversal of the generated electrical signal, these composites might be particularly well suited for sensors and for data storage, as only weak fields are required to delete or overwrite the information.

## Conflicts of interest

There are no conflicts to declare.

## Supplementary Material

RA-015-D5RA03466C-s001

## Data Availability

Data are available within the article or its SI. Supplementary information: X-ray diffraction patterns of all Fe_*x*_/(BaTiO_3_)_1−*x*_ composites after reduction and after sintering with carbon and zirconium carbide as oxygen getter. Gibbs free energy and corresponding equilibrium oxygen pressure for the systems FeO/Fe, CO_2_/CO and CO/C in the range 1300–1800 K. Comparison of Fe crystallite sizes of Fe_*x*_/(BaTiO_3_)_1−*x*_ composites sintered with C respectively ZrC as oxygen getter. Rietveld refinement of Fe_0.4_/(BaTiO_3_)_0.6_ (ZrC). High temperature impedance data of Fe_*x*_/(BaTiO_3_)_1−*x*_ samples. Field dependent magnetoelectric measurements of Fe_*x*_/(BaTiO_3_)_1−*x*_ for parallel and perpendicular sample set-up. Frequency dependence of the magnetoelectric coefficients of Fe_*x*_/(BaTiO_3_)_1−*x*_ composites sintered with ZrC as oxygen getter. See DOI: https://doi.org/10.1039/d5ra03466c.
