# Supplementary material for: Magnetoelectric properties of bulk 0-3 Fe/BaTiO3-composites
Source: RSC Adv. 2025 Sep 15;15(40):33480–93. doi: 10.1039/d5ra03466c (PMC12434467; doi:10.1039/d5ra03466c)
Supplement: RA-015-D5RA03466C-s001 [file RA-015-D5RA03466C-s001.pdf]

Supplementary Information (SI) for RSC Advances.
This journal is © The Royal Society of Chemistry 2025

Supporting Information

Magnetoelectric behavior of bulk 0-3 Fe/$BaTiO_3$-Composites

Toni Buttlar, Hartmut S. Leipner and Stefan G. Ebbinghaus*

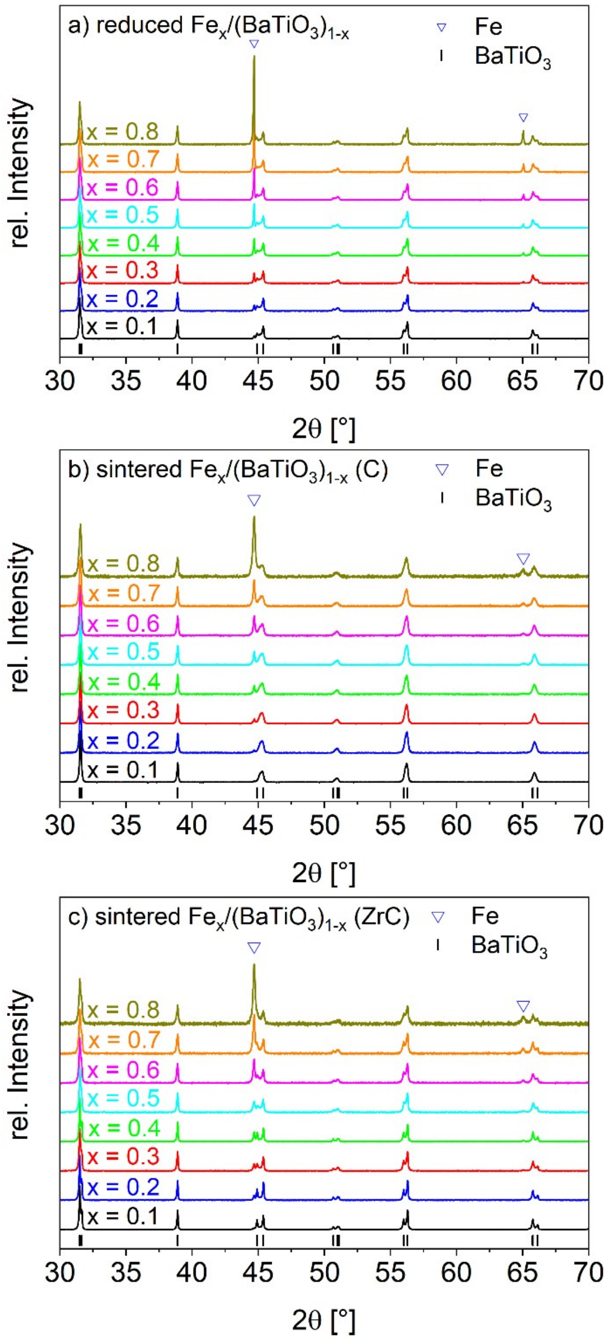

Figure S1: XRD pattern of $Fe_x/(BaTiO_3)_{1-x}$ (x = 0.1 - 0.8) after reduction in forming gas at 1073 K for 2 h (a) and after sintering in nitrogen at 1623 K for 2 h with carbon (b) and zirconium carbide as oxygen getter (c).

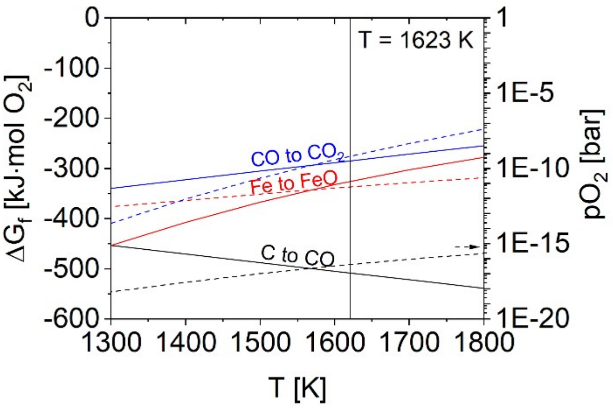

Figure S2: Gibbs free energy of the oxide formation for iron and carbon (full lines) and the corresponding equilibrium oxygen pressure (dashed lines) based on data of Barin et al.[1]

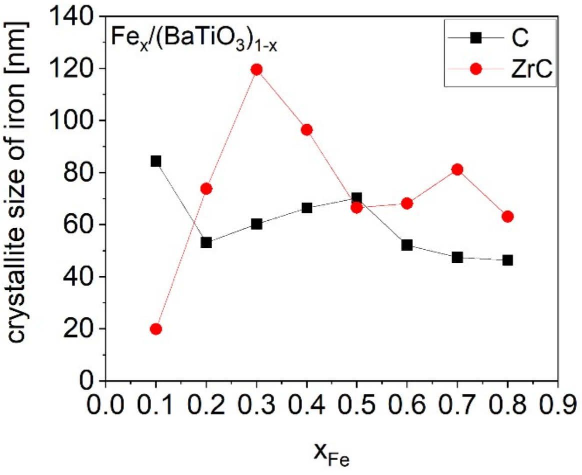

Figure S3: Comparison of Fe crystallite sizes of composites sintered with C (black) and with ZrC (red) as oxygen getter obtained from the diffraction peak at 44.8° as a function of the iron content. (lines are a guide to the eye)

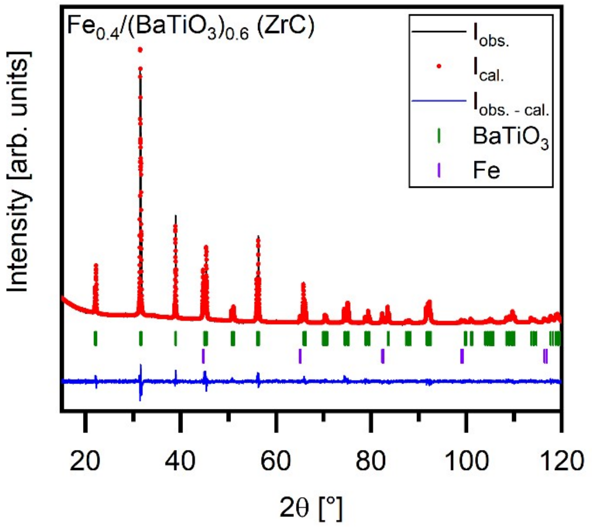

Figure S4: Rietveld refinement of $Fe_{0.4}/(BaTiO_3)_{0.6}$ after sintering in nitrogen at 1623 K for 2 h with zirconium carbide as oxygen getter.

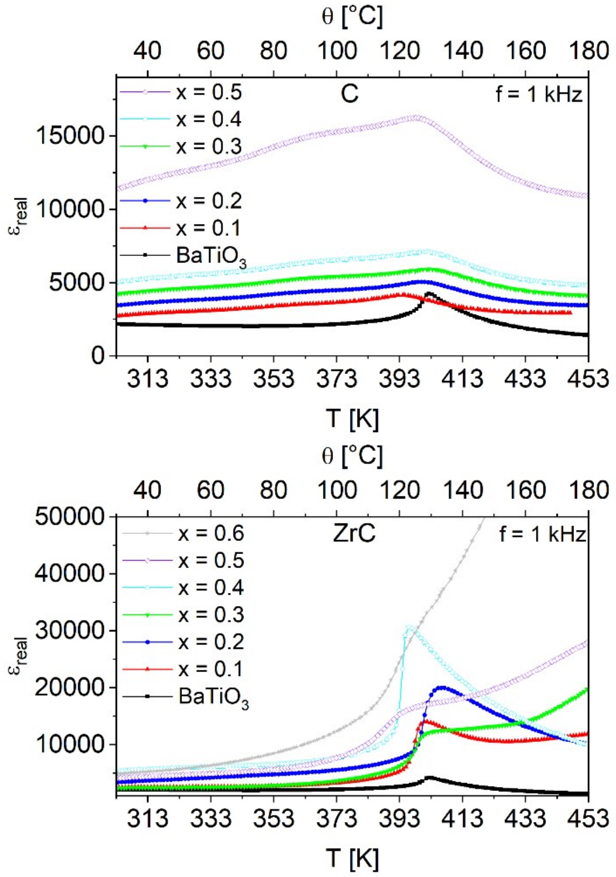

Figure S5: Impedance data of $Fe_x/(BaTiO_3)_{1-x}$ composites sintered with C (top, x = 0.1 - 0.5) and ZrC (bottom, x = 0.1 - 0.6) as oxygen getter in comparison to pure barium titanate (black).

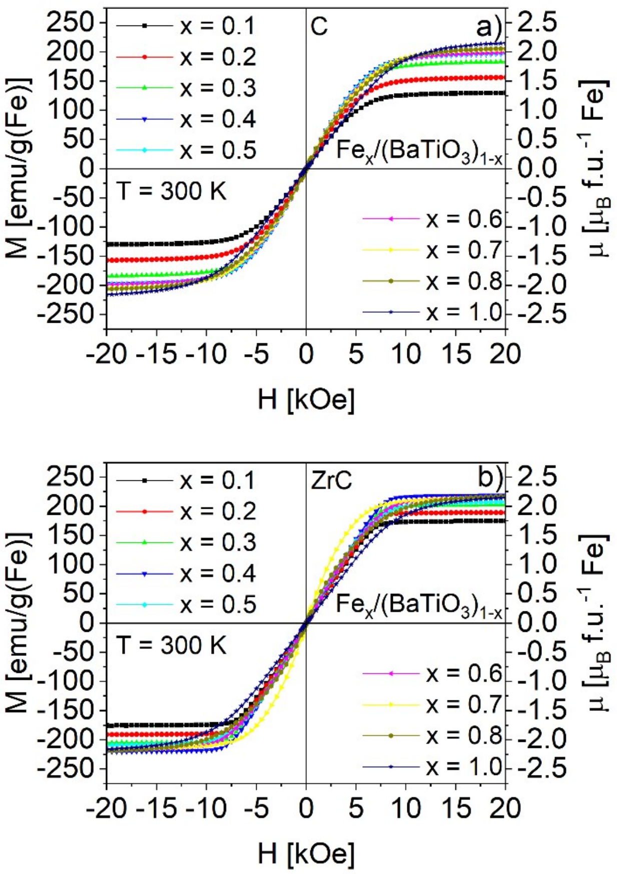

Figure S6: Field dependence of the magnetization of $Fe_x/(BaTiO_3)_{1-x}$ composites (x = 0.1 - 0.8) sintered with C (a) and ZrC (b) as oxygen getter (normalized to the nominal Fe content) in comparison to pure Fe.

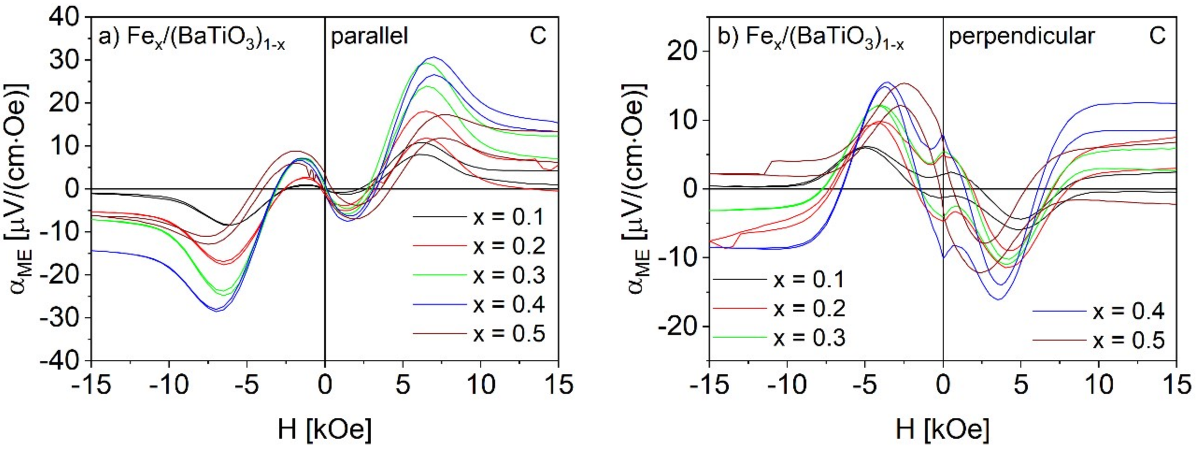

Figure S7: Influence of the magnetic DC field on the magnetoelectric coefficient of the $Fe_x/(BaTiO_3)_{1-x}$ samples (x = 0.1 to 0.5) sintered with C as oxygen getter for parallel (a)) and perpendicular orientation (b)).

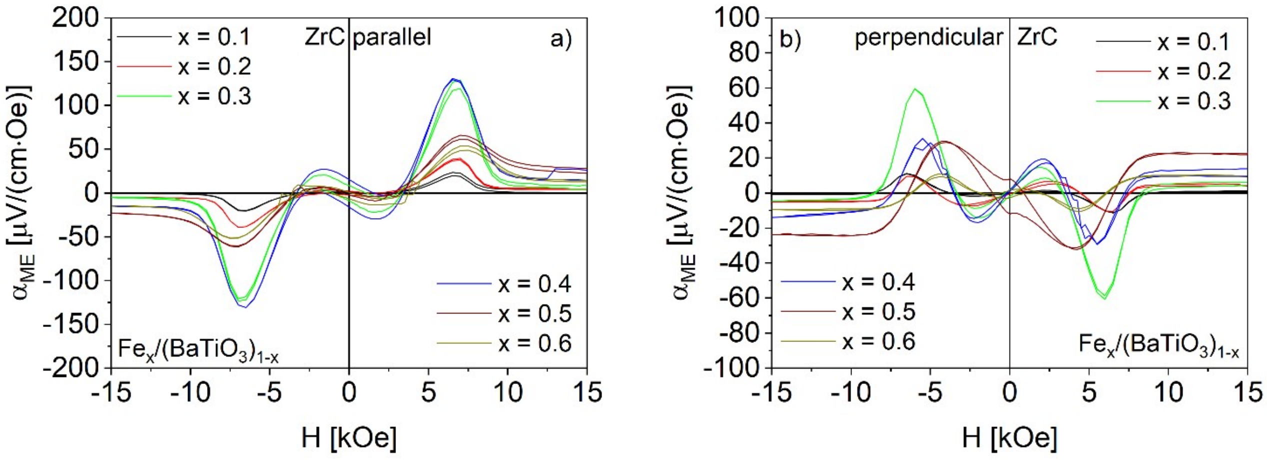

Figure S8: Influence of the magnetic DC field on the magnetoelectric coefficient of the $Fe_x/(BaTiO_3)_{1-x}$ samples (x = 0.1 to 0.6) sintered with ZrC as oxygen getter for parallel (a)) and perpendicular orientation (b)).

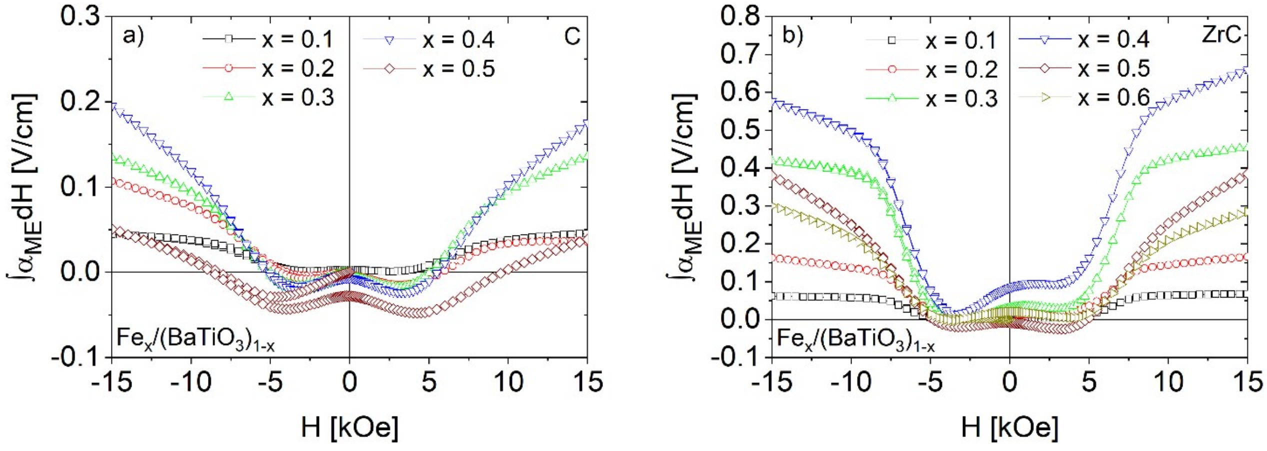

Figure S9: Comparison of the integration of α_{ME} of the $Fe_x/(BaTiO_3)_{(1-x)}$ composites sintered with C (a) and ZrC as oxygen getter (b).

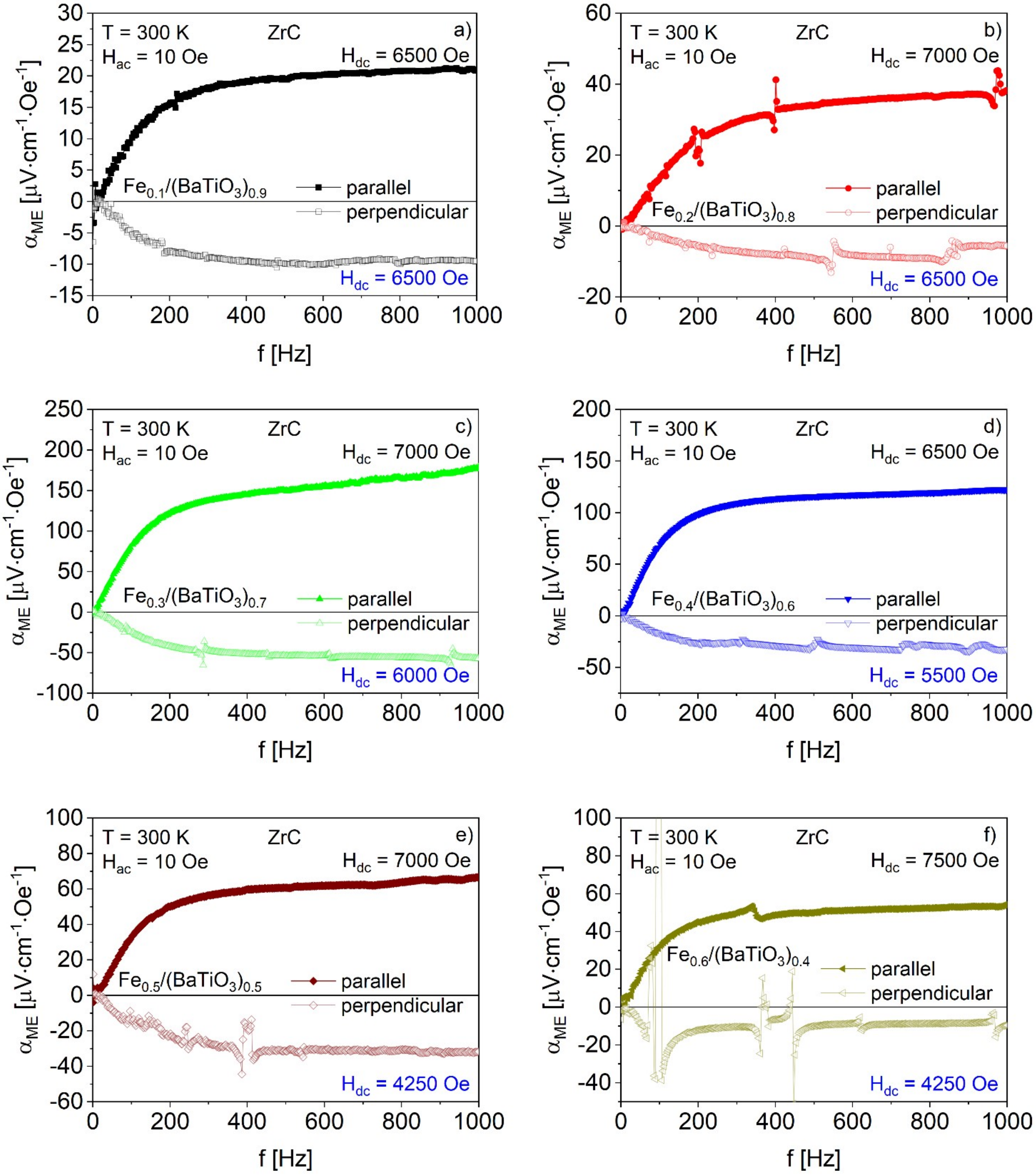

Figure S10: Influence of the magnetic ac-driving field frequency on α_{ME} for $Fe_x/(BaTiO_3)_{1-x}$ samples sintered with zirconium carbide as oxygen getter (x = 0.1 (a), 0.2 (b), 0.3 (c), 0.4 (d), 0.5 (e) and 0.6 (f)) in parallel (black filled squares) and perpendicular orientation (blue open squares).

References

1 I. Barin, *Thermochemical data of pure substances*, VCH, Weinheim, New York, 2008.
